# Supplementary material for: Anomalous metallic behaviour in the doped spin liquid candidate κ-(ET)4Hg2.89Br8
Source: Nat Commun. 2017 Oct 2;8:756. doi: 10.1038/s41467-017-00941-6 (PMC5624944; doi:10.1038/s41467-017-00941-6)
Supplement: Supplementary file 1 — Supplementary Information [file 41467_2017_941_MOESM1_ESM.pdf]

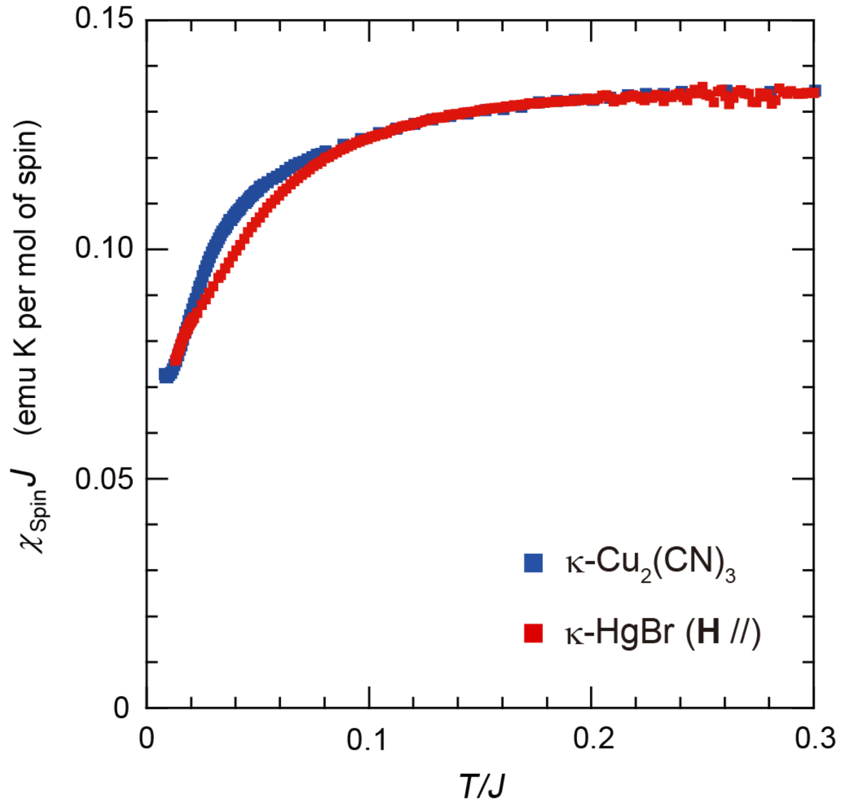

Supplementary Figure 1 │Scaled χ_{spin} of κ-$(ET)_4Hg_{2.89}Br_8$ and κ-$(ET)_2Cu_2(CN)_3$ (the low-temperature part of Fig. 2b). The slight kink visible at 0.02*J* (3 K) in κ-$(ET)_4Hg_{2.89}Br_8$ possibly comes from the superconducting diamagnetism, which is quite small under the parallel field of 1 T. Its T_c is reported to be 3.2 K at a magnetic field of 1 T[1].

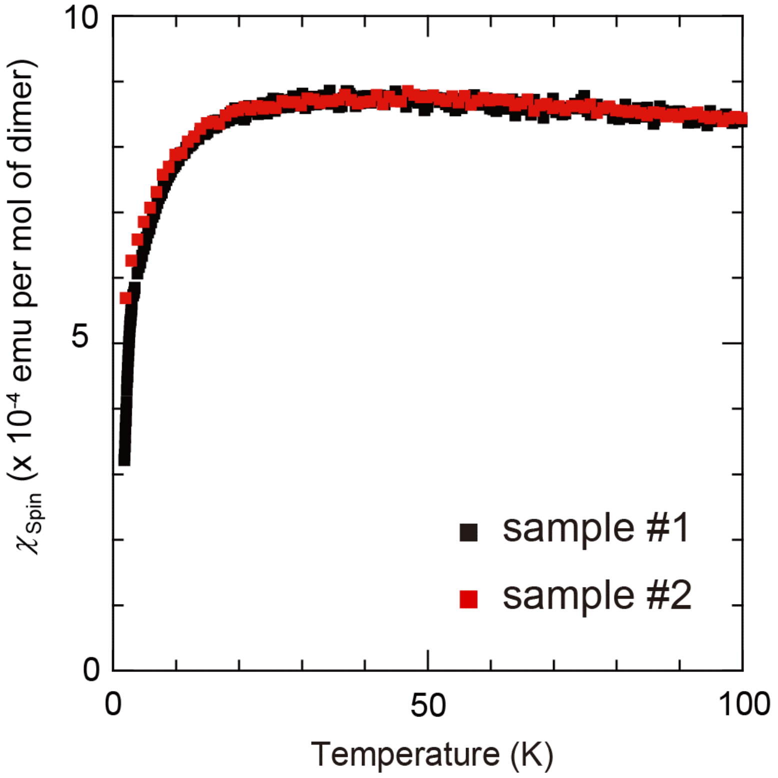

Supplementary Figure 2 │Sample dependence of χ_{spin} in κ-(ET)$_4$Hg$_{2.89}$Br$_8$. The #1 and #2 denote two separate crystals measured. A magnetic field of 1 T is applied perpendicular to the layers. In the normal state above 3 K, χ_{spin} is nearly sample-independent. A drop in χ_{spin} below 3 K in the sample #1 is due to a superconducting transition, the manifestation of which was sample-dependent.

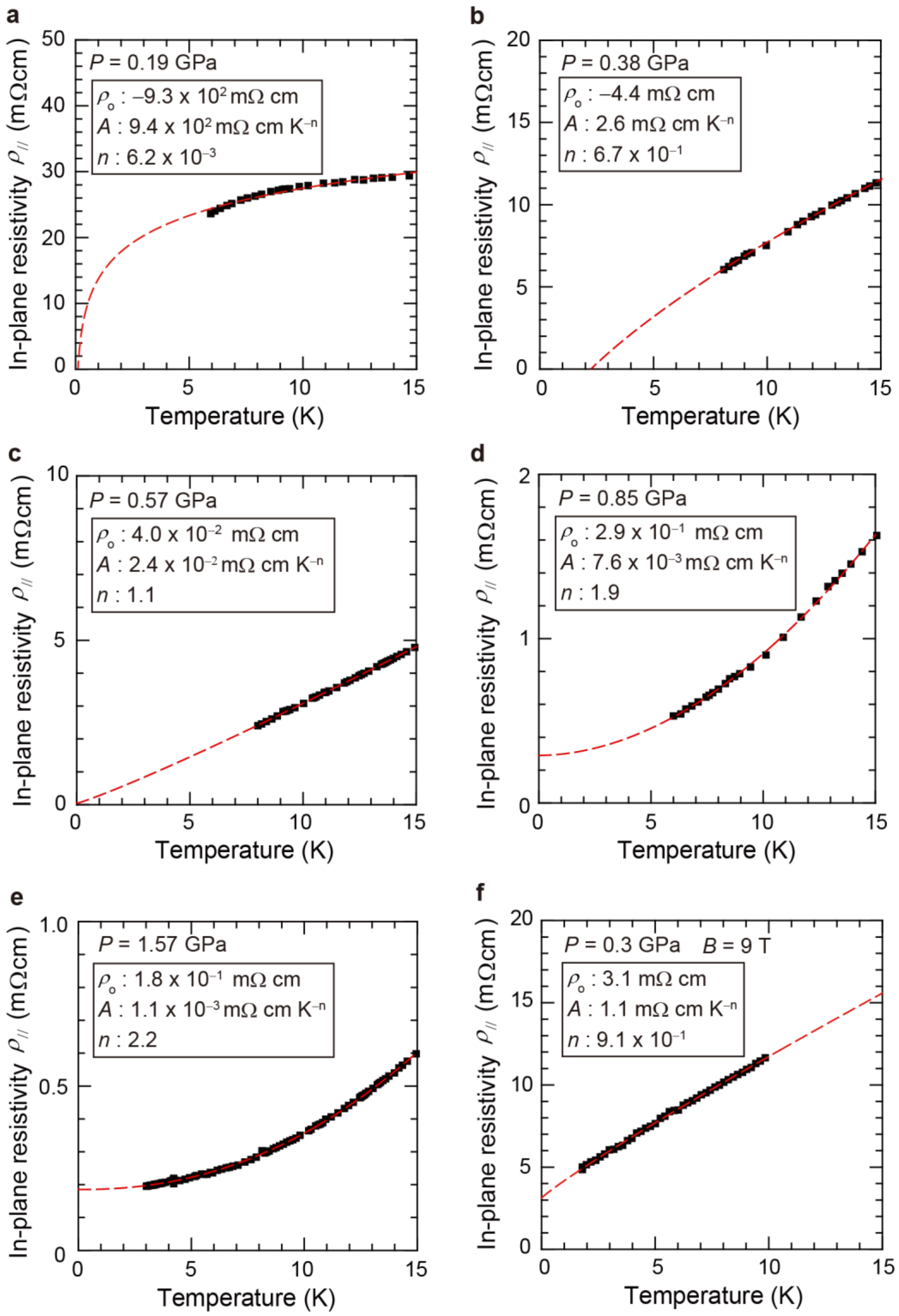

Supplementary Figure 3 │Power-law analysis of $\rho_{//}$. The functional form $\rho_{//} = \rho_o + AT^n$ is fitted to the resistivity data at 0.19 GPa (**a**), 0.38 GPa (**b**), 0.57 GPa (**c**), 0.85 GPa (**d**), 1.57 GPa (**e**), and 0.3 GPa (**f**). The resistivity data in (**a-e**) is the low temperature expansion of Fig. 3b, and that in (**f**) is presented in Fig. 5b.

Supplementary references

1. Lyubovskaya, R. N. *et al.* Anomalous dependence of the superconducting transition temperature in $(ET)_4Hg_{2.89}Br_8$ on the magnetic field and pressure. *Synth. Met.* **41-43**, 2147-2150 (1991).
